# Clinical significance of preoperative Glasgow prognostic score in patients with colorectal cancer and synchronous peritoneal metastases

**DOI:** 10.1002/ags3.12918

**Published:** 2025-01-24

**Authors:** Kosuke Fujimoto, Fumikazu Koyama, Hirotoshi Kobayashi, Kenjiro Kotake, Masayasu Kawasaki, Yukihide Kanemitsu, Yusuke Kinugasa, Hideki Ueno, Kotaro Maeda, Takeshi Suto, Michio Itabashi, Kimihiko Funahashi, Heita Ozawa, Shingo Noura, Hideyuki Ishida, Masayuki Ohue, Tomomichi Kiyomatsu, Soichiro Ishihara, Keiji Koda, Hideo Baba, Kenji Kawada, Yojiro Hashiguchi, Takanori Goi, Yuji Toiyama, Naohiro Tomita, Eiji Sunami, Fumihiko Fujita, Jun Watanabe, Kenichi Hakamada, Goro Nakayama, Kenichi Sugihara, Yoichi Ajioka

**Affiliations:** ^1^ Department of Surgery Nara Medical University Kashihara Japan; ^2^ Division of Endoscopy Nara Medical University Hospital Kashihara Japan; ^3^ Department of Surgery Tokyo Metropolitan Hiroo Hospital Tokyo Japan; ^4^ Department of Surgery Teikyo University Hospital Kawasaki Kanagawa Japan; ^5^ Department of Surgery Sano City Hospital Tochigi Japan; ^6^ Department of Surgery Bell Land General Hospital Sakai Japan; ^7^ Department of Colorectal Surgery National Cancer Center Hospital Tokyo Japan; ^8^ Department of Gastrointestinal Surgery Tokyo Medical and Dental University Tokyo Japan; ^9^ Department of Surgery National Defense Medical College Tokorozawa Japan; ^10^ International Medical Center Fujita Health University Hospital Toyoake Japan; ^11^ Department of Gastroenterological Surgery Yamagata Prefectural Central Hospital Yamagata Japan; ^12^ Department of Surgery, Institute of Gastroenterology Tokyo Women's Medical University Tokyo Japan; ^13^ Department of General and Gastroenterological Surgery Toho University Omori Medical Center Tokyo Japan; ^14^ Department of Surgery Tochigi Cancer Center Utsunomiya Japan; ^15^ Department of Surgery Osaka Rosai Hospital Sakai Japan; ^16^ Department of Digestive Tract and General Surgery, Saitama Medical Center Saitama Medical University Kawagoe Japan; ^17^ Department of Gastroenterological Surgery Osaka International Cancer Institute Osaka Japan; ^18^ Department of Colorectal Surgery National Center for Global Health and Medicine Tokyo Japan; ^19^ Department of Surgical Oncology The University of Tokyo Hospital Tokyo Japan; ^20^ Department of Surgery Teikyo University Chiba Medical Center Ichihara Japan; ^21^ Department of Gastroenterological Surgery, Graduate School of Medical Sciences Kumamoto University Kumamoto Japan; ^22^ Department of Gastrointestinal Surgery, Graduate School of Medicine Kyoto University Kyoto Japan; ^23^ Department of Surgery Kurashiki Central Hospital Kurashiki Okayama Japan; ^24^ Department of Surgery Teikyo University School of Medicine Tokyo Japan; ^25^ First Department of Surgery University of Fukui Fukui Japan; ^26^ Department of Gastrointestinal and Pediatric Surgery, Division of Reparative Medicine, Institute of Life Sciences Mie University Graduate School of Medicine Tsu Mie Japan; ^27^ Division of Lower Gastrointestinal Surgery, Department of Gastroenterological Surgery Hyogo Medical University Nishinomiya Hyogo Japan; ^28^ Department of Surgery Kyorin University School of Medicine Tokyo Japan; ^29^ Department of Surgery Kurume University School of Medicine Kurume Japan; ^30^ Department of Surgery, Gastroenterological Center Yokohama City University Medical Center Yokohama Japan; ^31^ Department of Colorectal Surgery Kansai Medical University Osaka Japan; ^32^ Department of Gastroenterological Surgery Hirosaki University Graduate School of Medicine Aomori Japan; ^33^ Department of Gastroenterological Surgery (Surgery II) Nagoya University Graduate School of Medicine Nagoya Japan; ^34^ Tokyo Medical and Dental University Tokyo Japan; ^35^ Division of Molecular and Diagnostic Pathology, Graduate School of Medical and Dental Sciences Niigata University Niigata Japan

**Keywords:** colorectal cancer, Glasgow prognostic score, indicators, peritoneal metastases, surgical resection

## Abstract

**Background:**

Chemotherapy is the typical choice for treating colorectal cancer with synchronous peritoneal metastases. Nonetheless, surgical resection may be chosen if the metastases are resectable. Unfortunately, there is no reliable preoperative or intraoperative prognostic indicator. This study aimed to determine the prognostic significance of the preoperative Glasgow prognostic score (GPS) in colorectal cancer patients with synchronous peritoneal metastases.

**Methods:**

We conducted a prospective study on 143 patients with colorectal cancer and concurrent peritoneal metastases. Our analysis included prognostic factors, such as the GPS, using data from the institutional observational study by the Japanese Society for Cancer of the Colon and Rectum.

**Results:**

The 3‐year survival rates for the GPS0 or 1 and GPS2 groups were 32.7% and 14.3%, respectively, with a significantly worse prognosis in the GPS2 group (*p* = 0.003). Multivariate analysis identified GPS2 (*p* = 0.006) and the peritoneal cancer index (PCI) (*p* = 0.029) or the Japanese surgical peritoneal metastasis grade (*p* = 0.009) as independent poor prognostic factors. Additionally, the GPS0 or 1 group with total resection of peritoneal metastases had a significantly better prognosis than the non‐resection group (*p* < 0.001); however, there was no difference between the GPS2 group with total peritoneal resection and the non‐resection group (*p* = 0.713).

**Conclusions:**

Preoperative GPS2 is an independent poor prognostic factor in patients with colorectal cancer and synchronous peritoneal metastases, and surgical resection does not improve prognosis in patients with GPS2. Preoperative GPSs may be used as indicators for surgical resection of synchronous peritoneal metastases.

## Graphical abstract

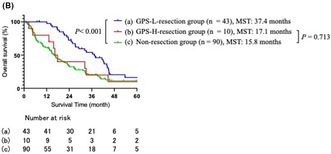

## 1 INTRODUCTION

Colorectal cancer (CRC) is the leading cause of cancer‐related death worldwide.^1^ In Japan, the incidence of CRC is increasing, and it is the second leading cause of cancer‐related deaths.^2^ In the treatment of advanced CRC with hematogenous metastases, if both the primary and metastatic lesions are resectable, the most promising treatment that contributes to the prognosis is surgical resection of all lesions, including primary and metastatic lesions.^3^, ^4^ Resection of liver and lung metastases is particularly effective, with reported 5‐year postoperative survival rates of 35%–58%^5^ and 30%–68%,^6^ respectively.

On the other hand, the prognosis of patients with peritoneal metastases is poor.^7^ The prevalence of synchronous peritoneal metastasis was reported to be 4%–8%.^8^, ^9^ Previously, the median survival time (MST) for patients with synchronous peritoneal metastases was 5–6 months.^8^ Advances in systemic chemotherapy have improved the MST for these patients to 12–33 months, but the 5‐year survival rate remains low, in the 0%–23% range.^9^ For this reason, peritoneal metastasis is classified as M1c in both the Union for International Cancer Control (UICC) TNM classification 8th edition and the Japanese Classification of Colorectal, Appendiceal, and Anal Carcinoma, the 3rd English Edition, and the stages are classified as IVC and IVc, respectively.^10^, ^11^

Regarding treatment strategies for synchronous peritoneal metastasis, chemotherapy is the first‐line treatment strategy when they are clearly considered unresectable.^3^ Given that primary tumor resection followed by chemotherapy showed no survival benefit over chemotherapy alone, primary tumor resection should no longer be considered a standard of care for patients with CRC with asymptomatic primary tumors and synchronous unresectable metastases.^12^ Recently, several studies have reported 5‐year survival rates of 19%–51% in selected patients with peritoneal metastases treated with a combination of perioperative systemic chemotherapy and cytoreductive surgery with or without hyperthermic intraperitoneal chemotherapy (HIPEC).^13^ This treatment is currently listed as a treatment option in the European Society for Medical Oncology (ESMO) and National Comprehensive Cancer Network (NCCN) guidelines.^3^, ^4^ However, the number of patients who can actually receive this treatment is limited because it is highly invasive and available only at a few specialized facilities.^13^ For this reason, many clinicians choose chemotherapy for patients with multiple peritoneal metastases that are clearly visible on preoperative imaging and whose primary tumors are asymptomatic.

Peritoneal metastases can also be observed during surgery. In such cases, an intraoperative decision must be made to resect the peritoneal metastases along with the primary tumor. Improved prognosis and long‐term survival have been reported after total lesion resection in patients with localized peritoneal metastases.^14^ Thus, surgical resection may be an option for simultaneous localized peritoneal metastases that can be resected without excessive invasion.^15^ However, no useful indicator has been found to determine which patients are expected to have an improved prognosis if surgical resection is indicated. In addition, at the time of surgery, decisions must be made without detailed pathological information.

Recently, accumulating evidence has shown that systemic inflammatory response and nutritional status are associated with prognoses in cancer patients.^16^ These indices, including the Glasgow prognostic score (GPS),^17^ neutrophil/lymphocyte ratio,^18^ and prognostic nutritional index (PNI),^19^ have been reported. The GPS is an index that simultaneously evaluates systemic inflammation and nutritional status by scoring the cutoff values of serum albumin and C‐reactive protein (CRP) levels as 3.5 g/dL and 1.0 mg/dL, respectively.^17^ The GPS can be easily evaluated preoperatively and can be used for preoperative and intraoperative treatment decisions. If the GPS can predict the prognosis of patients with CRC and peritoneal metastases, it can be an important indicator to better determine the indications for surgical resection, avoid unnecessarily invasive procedures, and lead more quickly to appropriate treatment, such as chemotherapy.

The purpose of this prospective study was twofold. First, we aimed to determine the prognostic impact of the GPS in patients with CRC undergoing surgery for CRC with synchronous peritoneal metastases. Second, we aimed to determine whether the GPS could help determine the indications for complete resection of peritoneal metastases.

## 2 METHODS

### 2.1 Study design

The 28 member hospitals of the Japanese Society for Cancer of the Colon and Rectum (JSCCR) were included in this multi‐institutional prospective observational study and enrolled patients who underwent surgery for CRC with synchronous peritoneal metastasis between October 2012 and December 2016.

The protocol for this research project has been approved by a suitably constituted Ethics Committee of the JSCCR and each institution, and it conforms to the ethical guidelines of the 2008 Declaration of Seoul. Written informed consent was obtained from all patients before enrollment. As synchronous peritoneal metastases from CRC are often found accidentally during surgery, written informed consent could be obtained after surgery in such cases. Clinical and pathological information was registered within 3 months after surgery. Prognostic information was collected 3 years after surgery.

### 2.2 Surgical procedure

The surgical procedure was not determined by the protocol used in this study because of the variety of conditions in patients with synchronous peritoneal metastases. Each surgeon made a decision regarding resection of the primary tumor and peritoneal metastases.

### 2.3 Data collection

All data were prospectively collected. Preoperative data included physical information, blood tests, and preoperative diagnoses. Information regarding peritoneal metastases included both the Japanese classification and the peritoneal cancer index (PCI) score.^11^, ^20^ The size and number of peritoneal metastases in each region were recorded. Information regarding surgical procedures and other pathological information were sent within 3 months after the surgery. Information regarding postoperative chemotherapy and prognoses was collected at least 3 years after surgery.

### 2.4 Japanese classification

The Japanese classification of colorectal carcinoma is published by the JSCCR.^11^ In the present Japanese classification, distant metastases and synchronous peritoneal metastases were classified as follows:

M0: No distant metastasis.

M1a: Distant metastasis confined to one organ. Peritoneal metastasis not present.

M1b: Distant metastasis in more than one organ. Peritoneal metastasis not present.

M1c1: Metastasis to the peritoneum only.

M1c2: Metastasis to the peritoneum with other distant metastases.

P0: No peritoneal metastasis.

P1: Metastasis localized to adjacent peritoneum.

P2: Limited metastasis to distant peritoneum.

P3: Diffuse metastasis to distant peritoneum.

The categories of findings, namely clinical, surgical, and pathological findings, are identified by placing the lowercase letters “c,” “s,” and “p,” respectively, in front of each uppercase letter.

### 2.5 Estimation of GPS

The GPS was assessed preoperatively: patients with both elevated CRP levels (>1.0 mg/dL) and hypoalbuminemia (<3.5 g/dL) received 2 points, those with only one of these biochemical abnormalities received 1 point, and those with neither received 0 points.^17^ In this study, GPS0 and 1 were defined as GPS‐low (L) and GPS2 as GPS‐high (H).

### 2.6 Statistical analyses

Results are expressed as numbers and percentages or as means with standard deviations (SDs). Statistical significance was evaluated using the chi‐squared, Fisher's exact, or Student's *t*‐tests. Overall survival (OS) was defined as the time from the date of surgery to death. The Kaplan–Meier method was used to estimate survival curves, and the log‐rank test was performed to evaluate differences in survival curves. A Cox proportional hazards model was used for the univariate and multivariate survival analyses. The multivariate analysis included significant variables in the univariate analysis and variables considered clinically associated with OS among the preoperative and intraoperative factors. All *p*‐values <0.05 were considered significant, and 95% confidence intervals (CIs) were calculated. SPSS software (version 22.0; SPSS, Chicago, IL, USA) was used for statistical analyses.

## 3 RESULTS

In total, 150 patients were enrolled in this study. We excluded seven patients for whom serum albumin and CRP levels had not been measured preoperatively. Thus, 143 patients were included in this study. Table 1 presents the patient characteristics of the entire cohort. The median age of the patients was 66 years. The primary tumor was located in the right colon in approximately half of the patients. According to the present Japanese classification, the number of patients with synchronous peritoneal metastases was 29 (20%) with P1, 54 (38%) with P2, and 60 (42%) with P3. The median PCI score was 4 (1–29) in this study. Fifty‐nine patients (41%) had other synchronous metastases. Nine (6.3%) patients received preoperative chemotherapy.

**TABLE 1:** Patients' characteristics.

Variables		
Age (years)	<66 ≥66	70 73
Gender	Male Female	81 62
Performance status	<2 ≥2	133 10
Hypertension	Positive Negative	106 37
Diabetes mellitus	Positive Negative	124 19
Cardiovascular disease	Positive Negative	129 14
Location of primary tumor	Left side Right side	69 74
Location of primary tumor	Rectum Colon	26 117
cT‐category^a^	<cT4a cT4b Unknown	118 24 1
cN‐category^a^	<cN2 ≥cN2 Unknown	77 64 2
cM‐category^a^	cM1a/cM1b cM1c	129 14
CEA (ng/mL)	<5 ≥5 Unknown	34 108 1
CA19‐9 (U/mL)	<37 ≥37 Unknown	67 75 1
ALB	≥3.5 <3.5	83 60
CRP	≤1.0 >1.0	82 61
GPS	Low High	101 42
Preoperative chemotherapy	Absent Present	134 9
sT‐category^a^	<sT4a sT4b Unknown	95 42 6
sM‐category^a^	sM1c1 sM1c2	59 84
PCI	≤4 >4	76 67
sP‐grade^a^	≤sP2 sP3	83 60

Abbreviations: ALB, serum albumin level; CRP C‐reactive protein level; GPS Glasgow prognostic score; PCI peritoneal cancer index.

^a^ The Japanese classification of colorectal carcinoma published by the Japanese Society for Cancer of the Colon and Rectum. T and N‐category were same as the UICC TNM classification. See the text for M‐category and P‐grade.

### 3.1 Glasgow prognostic scores

Of all the patients, 64 had GPS0, 37 had GPS1, and 42 had GPS2. In this study, we divided the patients into two groups: the GPS‐low (L) group with GPS0 or 1 and the GPS‐high (H) group with GPS2.

### 3.2 Overall survival

**FIGURE 1:**
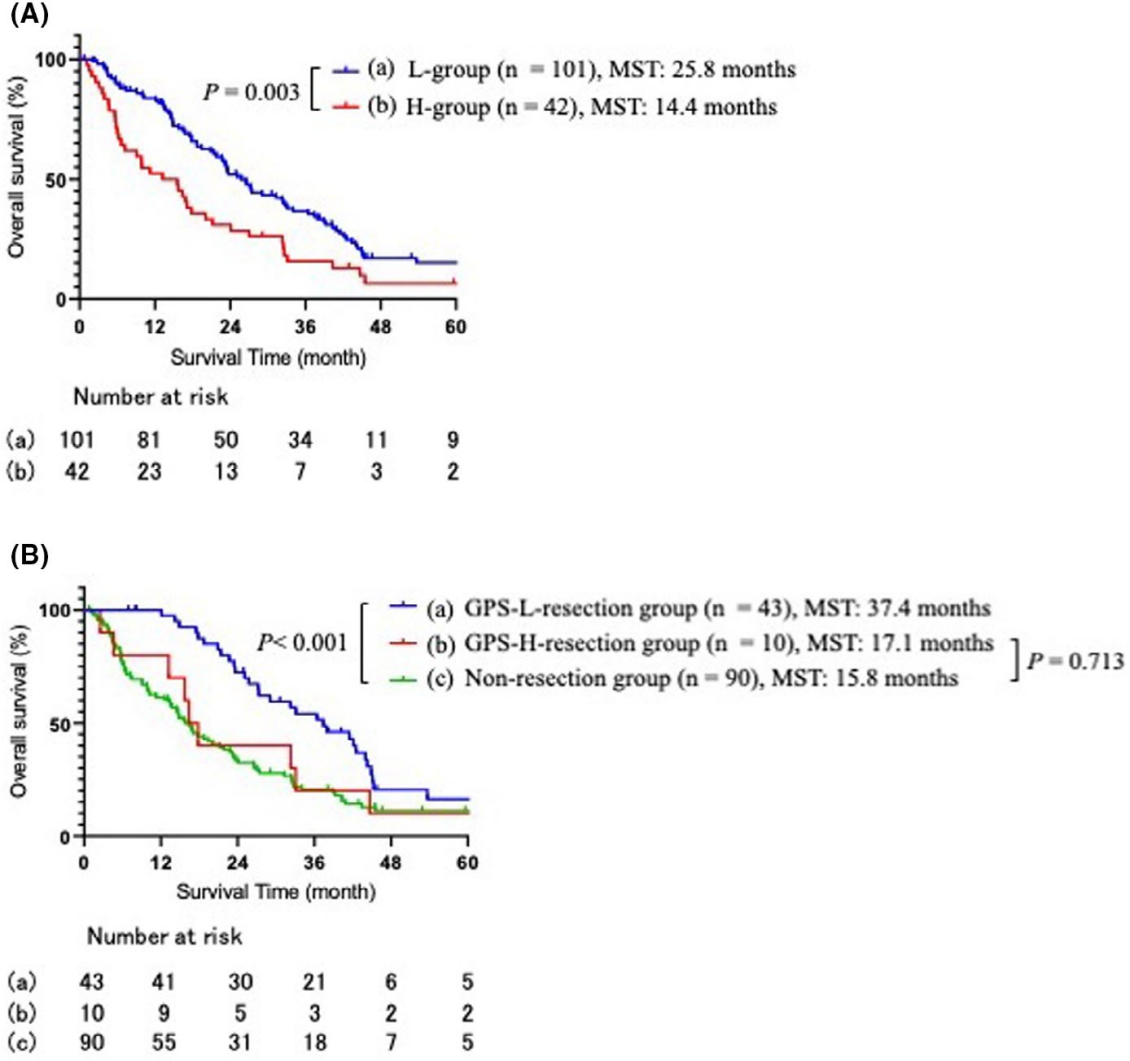
(A) Survival curves for the L‐group and H‐group, respectively. (B) Survival curves for the L‐resection group, H‐resection group, and non‐resection group, respectively.

The entire cohort's MST was 22.8 months. The MST of the L‐group and H‐group was 25.8 and 14.0 months, respectively (*p* = 0.003, Figure 1A). The 3‐year survival rates in the L‐group and H‐group were 32.7% and 14.2%, respectively.

### 3.3 Predictive factor for OS

In the univariate analysis of the OS, the HR for GPS2 was 1.945 (95% CI, 1.247–3.033; *p* = 0.003). Other factors that significantly correlated with OS were M1c2 (*p* = 0.033), P3 (*p* = 0.001), and PCI ≥4 (*p* = 0.013). Due to confounding between the PCI and surgical peritoneal metastasis grade (sP1‐3) according to the Japanese classification, multivariate analyses were performed separately for each. The multivariate analysis demonstrated that GPS2 (*p* = 0.016, 0.005), sP3 (*p* = 0.009), and PCI ≥4 (*p* = 0.029) were independent predictors for the OS (Table 2).

**TABLE 2:** The results of the univariate and multivariate analysis of the factors associated the overall survival.

Variables		Univariate	Multivariate	
		*p* value	Hazard ratio (95% CI)	*p* value
Age (years)	<66 ≥66	0.450		
Gender	Male Female	0.182		
Performance status	<2 ≥2	0.428		
Hypertension	Positive Negative	0.685		
Diabetes mellitus	Positive Negative	0.299		
Cardiovascular disease	Positive Negative	0.158		
Location of primary tumor	Left side Right side	0.705		
Location of primary tumor	Rectum Colon	0.098		
cT‐category^a^	<cT4a cT4b	0.077		
cN‐category^a^	<cN2 ≥cN2	0.853		
cM‐category^a^	cM1a/cM1b cM1c	0.786		
CEA (ng/mL)	<5 ≥5	0.080		
CA19‐9 (U/mL)	<37 ≥37	0.058		
ALB (g/dL)	≥3.5 <3.5	0.265		
CRP (mg/dL)^b^	≤1.0 >1.0	0.002		
GPS	Low High	0.003	1 1.721 (1.165–2.544)	0.006^c^
Preoperative chemotherapy	Absent Present	0.094		
sT‐category^a^	<sT4a sT4b	0.192		
sM‐category^a^	sM1c1 sM1c2	0.033	1 1.415 (0.972–2.062)	0.070^c^
PCI	≤4 >4	0.013	1 1.505 (1.043–2.173)	0.029^c^
sP‐grade	≤sP2 sP3	0.001	1 1.658 (1.136–2.419)	0.009^d^

Abbreviations: ALB, serum albumin level; CA19‐9, carbohydrate antigen 19‐9; CEA, carcinoembryonic antigen; CI, confidence interval; CRP, C‐reactive protein level; GPS, Glasgow prognostic score; PCI, peritoneal cancer index.

^a^ The Japanese classification of colorectal carcinoma published by the Japanese Society for Cancer of the Colon and Rectum. T‐category and N‐category were same as the UICC TNM classification. See the text for the M‐category and P‐grade.

^b^ As both CRP are constituent elements of the GPS score, we deliberately excluded them from the multivariate analysis to avoid multicollinearity.

^c^ Simultaneously adjusted for GPS, sM‐category, and PCI.

^d^ Simultaneously adjusted for GPS, sM‐category, and sP‐grade.

### 3.4 Relationship between patient characteristics and the GPS

The relationship between the clinicopathological characteristics of the patients and GPS is shown in Tables 3 and 4. The mean age was significantly higher in the H‐group than in the L‐group (*p* < 0.001). The H‐group had significantly poorer performance statuses (PSs) than the L‐group (*p* = 0.037). The rate of adjuvant chemotherapy initiation among patients who underwent R0 resection was 80.0% (24/30) in the L‐group and 80.0% (4/5) in the H‐group (*p* = 0.697). The induction rate of first‐line chemotherapy in patients who underwent R2 resection was 86.3% (63/73) in the L‐group and 76.9% (30/39) in the H‐group (*p* = 0.208), and the proportion of patients who received chemotherapy using multiple drugs was 87.3% in the L‐group and 80% in the H‐group (*p* = 0.357). The median number of courses of first‐line chemotherapy administered was 10.5 in the L‐group and 8.5 in the H‐group, with no significant differences (*p* = 0.357). Regarding the extent of peritoneal metastases, the H‐group had a significantly higher proportion of P3 (*p* = 0.006) and significantly higher mean PCI score (*p* = 0.013) than the L‐group. More patients in the L‐group underwent resection of the primary tumor (*p* = 0.034) and total resection of peritoneal metastases (*p* = 0.032) than those in the H‐group.

**TABLE 3:** The relationship between GPS and the clinicopathological characteristics of the patients.

Variables		Low GPS (*n* = 101, %)	High GPS (*n* = 42, %)	*p* value
Age (years)	<66 ≥66	59 (58.4) 42 (41.6)	11 (26.2) 31 (73.8)	<0.001
Gender	Male Female	58 (57.4) 43 (42.6)	23 (54.8) 19 (45.2)	0.770
Performance status	<2 ≥2	97 (96.0) 4 (4.0)	36 (85.7) 6 (14.3)	0.037
Hypertension	Positive Negative	80 (79.2) 21 (20.8)	26 (61.9) 16 (38.1)	0.031
Diabetes mellitus	Positive Negative	89 (88.1) 12 (11.9)	35 (83.3) 7 (16.7)	0.433
Cardiovascular disease	Positive Negative	93 (92.1) 8 (7.9)	36 (85.7) 6 (14.3)	0.193
Location of primary tumor	Left side Right side	48 (47.5) 53 (52.5)	21 (50.0) 21 (50.0)	0.787
cT‐category^a^, ^b^	<cT4a cT4b	87 (87.0) 13 (13.0)	31 (73.8) 11 (26.2)	0.056
cN‐category^a^, ^c^	<cN2 ≥cN2	53 (53.5) 46 (46.5)	24 (57.1) 18 (42.9)	0.694
sM‐category^a^	sM1c1 sM1c2	44 (43.6) 57 (56.4)	15 (35.7) 27 (64.3)	0.385
CEA (ng/mL)	<5 ≥5	28 (27.7) 73 (72.3)	6 (14.6) 35 (85.4)	0.098
CA19‐9 (U/mL)	<37 ≥37	52 (51.5) 49 (48.5)	15 (36.6) 26 (63.4)	0.107

Abbreviations: CEA, carcinoembryonic antigen; GPS, Glasgow prognostic score; CA19‐9, carbohydrate antigen 19‐9.

^a^ The Japanese classification of colorectal carcinoma published by the Japanese Society for Cancer of the Colon and Rectum. T‐category and N‐category were same as the UICC TNM classification. See the text for the M‐category and P‐grade.

^b^ There was one case of missing data in the cT category for the low GPS group.

^c^ There were two cases of missing data in the cN category for the low GPS group.

**TABLE 4:** The relationship between GPS and perioperative data.

Variables		Low GPS (*n* = 101, %)	High GPS (*n* = 42, %)	*p* value
sP‐grade^a^	<sP3 sP3	66 (65.3) 35 (34.7)	17 (40.5) 25 (59.5)	0.006
PCI		6.8^d^ ± 6.6^e^	10.1^d^ ± 8.2^e^	0.013
Resection of primary lesion	Absent Present	12 (11.9) 89 (88.1)	11 (26.2) 31 (73.8)	0.034
Total resection of peritoneal metastases	Absent Present	58 (57.4) 43 (42.6)	32 (76.2) 10 (23.8)	0.034
Surgical curability	R0 R1, R2	29 (28.7) 72 (71.3)	5 (11.9) 37 (88.1)	0.032
pT‐category^a^, ^b^	<pT4a pT4b	72 (80.9) 17 (19.1)	22 (71.0) 9 (29.0)	0.307
pN‐category^a^, ^b^	<pN2 ≥pN2	52 (58.4) 37 (41.6)	15 (48.4) 16 (51.6)	0.276
Venous invasion^a^, ^b^	<V1a ≥V1a	4 (4.5) 85 (95.5)	3 (9.7) 27 (90.3)	0.376
Lymphatic invasion^a^, ^b^	<Ly1b ≥Ly1b	13 (14.6) 76 (85.4)	4 (12.9) 27 (87.1)	0.582
Cytology of ascites^c^	Negative Positive	23 (22.8) 33 (32.7)	7 (16.7) 14 (33.3)	0.535
Operative time (min)		214.6^d^ ± 112.4^e^	177.9^d^ ± 87.2^e^	0.061
Blood loss (mL)		231.5^d^ ± 385.7^e^	370.4^d^ ± 475.4^e^	0.070

Abbreviations: GPS, Glasgow prognostic score; PCI, peritoneal cancer index.

^a^ The Japanese classification of colorectal carcinoma published by the Japanese Society for Cancer of the Colon and Rectum. T‐category and N‐category were same as the UICC TNM classification. See the text for the M‐category and P‐grade. V0, no lymphatic invasion, V1a, minimal lymphatic invasion, V1b, moderate lymphatic invasion, V1c, severe lymphatic invasion. Ly0, no lymphatic invasion, Ly1a, minimal lymphatic invasion, Ly1b, moderate lymphatic invasion, Ly1c, severe lymphatic invasion.

^b^ Twelve cases in the low‐GPS group and 11 cases in the high GPS group did not undergo primary tumor resection. Additionally, among the cases that underwent primary tumor resection in the high GPS group, there was one case of missing data for venous invasion evaluation.

^c^ In 66 cases, ascites cytology was not performed.

^d^ Mean.

^e^ Standard deviation.

In addition, we investigated the prognoses of patients in the L‐group who underwent total resection of peritoneal metastases (L‐resection group), those in the H‐group who underwent total resection of peritoneal metastases (H‐resection group), and those who did not undergo total peritoneal metastases resections (non‐resection group). The L‐resection group had a significantly better prognosis than the non‐resection group (*p* < 0.001), whereas the prognosis of the H‐resection group did not differ from that of the non‐resection group (*p* = 0.713) (Figure 1B). We also compared resected and non‐resected groups within the H‐group. There was no significant difference in prognosis between the two groups (*p* = 0.367).

## 4 DISCUSSION

Identifying the prognostic factors for OS is important for the selection and planning of treatments for patients with cancer. However, studies examining prognostic factors in patients with CRC and concurrent peritoneal metastases are limited. To date, the PCI, cytoreductive integrity score, serum carcinoembryonic antigen (CEA) and carbohydrate antigen 19‐9 (CA19‐9) levels, regional lymph node metastases, and distant metastases other than peritoneal metastases have been reported as prognostic factors.^21^, ^22^, ^23^

The GPS is a scoring system proposed by Donald C. McMillan, which combines serum CRP and albumin levels.^17^ CRP is a protein produced by interleukin‐6 and other inflammatory cytokines acting on the liver.^24^ Therefore, as cancer progresses and the number of cancer cells increases, inflammatory cytokines produced by the cancer cells themselves or by lymphocytes sensitized by the cancer cells increase in the blood. As a result, CRP levels increase. Inflammatory cytokines inhibit protein synthesis in muscle tissues and promote protein decay. As a result, the Alb values decrease. Thus, the GPS is said to reflect the pathophysiology of malnutrition based on a systemic inflammatory response in patients with cancer.^25^ The GPS was first reported as a prognostic factor for non‐small‐cell lung cancer. Later, it was shown to be a prognostic factor independent of clinical stage, not only in various primary malignancies but also in metastatic tumors of breast cancer, renal cell carcinoma, and esophageal cancer.^26^ In CRC, the GPS has been reported to be a useful prognostic factor not only in cases of curative resection at Stages I–III but also in cases of resection of liver metastases and unresectable advanced or recurrent disease.^27^ These findings suggest that the GPS may be a prognostic factor in patients with CRC and synchronous peritoneal metastases. Indeed, in a single‐center retrospective study, Adachi et al. found that the modified GPS was associated with 3‐month postoperative mortality in patients with CRC and synchronous peritoneal metastases.^28^

The primary objective of this multicenter prospective study was to determine the prognostic impact of the GPS in patients with CRC who underwent surgery for CRC with synchronous peritoneal metastases. To ultimately contribute to the decision on the indication for surgery, we evaluated patients with CRC who underwent surgery for CRC and synchronous peritoneal metastases (including non‐resection), regardless of R0 resection, and examined the prognostic factors using preoperative and intraoperative factors, including the GPS. Our results clearly demonstrate, for the first time in a prospective study, that GPS2 is a poor prognostic factor in patients with simultaneous peritoneal metastases.

The second objective of this study was to determine whether the GPS can help determine the indications for complete resection of peritoneal metastases. Patients with a GPS of 0 or 1 who underwent complete resection of peritoneal metastases had a significantly better prognosis than those who did not undergo complete resection. In contrast, patients with a GPS of 2 who underwent complete resection of peritoneal metastases had no difference in prognosis compared to patients who did not undergo complete resections. This suggests that patients with CRC peritoneal metastases with GPSs of 0 and 1 can be expected to have an improved prognosis with surgical resection of all lesions, whereas patients with GPSs of 2 cannot be expected to benefit from surgical resection.

In this study, sP3 and PCI ≥4 were identified as independent poor prognostic factors in addition to the GPS in the multivariate analysis of prognoses. These findings validate the three categories of peritoneal metastases^11^ and are consistent with previous reports that the PCI correlates with prognosis in patients with peritoneal metastases.^22^, ^23^ In addition, an association analysis of GPS with patient characteristics showed that the GPS was associated with the PCI, PS, age, and hypertension. The PCI is an objective measure of the tumor burden of peritoneal metastases,^20^ and the GPS may reflect the tumor burden, including the degree of peritoneal metastases. The PS is an indicator of activities of daily living (ADL). An association between PS and the GPS has been reported in metastatic pancreatic cancer,^29^ and the GPS may also reflect patient ADLs in peritoneal metastases of CRC. The relationship between the GPS and age or hypertension may reflect a patient's underlying condition other than CRC progression. In a previous study, Shida et al. reported that distant metastasis, the PCI, and PS were the three factors influencing R0 resection in M1c disease.^30^ In other words, the GPS may be used as a comprehensive index for treatment selection in patients with CRC peritoneal metastases, encompassing not only the progression of peritoneal metastases but also the patients' ADL and the possibility of R0 resection.

This study had some limitations. First, as in previous reports, the GPS was associated with age and PS in this study, but there was neither difference in the rate of chemotherapy induction between patients with high and low GPSs, nor in the choice of drug or number of courses used. Usually, patients with PS3 or higher are not eligible for systemic drug therapy, but in this study, 0% of patients had PS3 or higher. This is because the study included patients who were originally targeted for aggressive treatment, including surgery. This is one limitation in interpreting the results of this study. Second, in this study, detailed information regarding chemotherapy response rates and reasons for regimen modifications was not available. While our findings suggest that the GPS reflects disease status, we were unable to fully discuss the relationship between the GPS and chemotherapy as a prognostic factor. Although there were no significant differences between the L‐group and H‐group in terms of adjuvant chemotherapy initiation rates and the number of first‐line regimen cycles administered, we acknowledge that we could not evaluate the effectiveness of or resistance to individual chemotherapy regimens. This represents an important limitation of our analysis. Third, our study lacked genetic testing information, including microsatellite instability status, RAS mutation, and BRAF mutation. These molecular characteristics could potentially influence both patient prognosis and treatment strategies in CRC with peritoneal metastases. In addition, because the enrollment period was a bit old (2012–2016), the survival of patients diagnosed with peritoneal metastases preoperatively may differ between then and now due to advances in drug therapy. However, oxaliplatin, irinotecan, bevacizumab, cetuximab, and panitumumab were already available in Japan in 2012, and the main progress in chemotherapy since then has been the improvement of late‐line therapies. Therefore, the impact of advances in chemotherapy during this period on the initial treatment strategy for patients diagnosed with peritoneal metastases is not expected to be significant.

In conclusion, this is the first prospective study to demonstrate that preoperative GPS is a prognostic factor in patients undergoing surgery for CRC with peritoneal metastases. In the treatment selection of patients with CRC and simultaneous peritoneal metastases, surgical resection of all lesions may improve the prognosis in the cases of GPSs of 0 and 1, but not in the case of GPSs of 2.

## AUTHOR CONTRIBUTIONS

**Kosuke Fujimoto:** Conceptualization; formal analysis; investigation; methodology; visualization; writing – original draft; writing – review and editing. **Fumikazu Koyama:** Conceptualization; data curation; formal analysis; investigation; methodology; project administration; visualization; writing – review and editing. **Hirotoshi Kobayashi:** Conceptualization; data curation; funding acquisition; investigation; methodology; project administration; resources; software; supervision; validation; writing – review and editing. **Kenjiro Kotake:** Conceptualization; data curation; funding acquisition; investigation; methodology; project administration; resources; software; supervision; validation; writing – review and editing. **Masayasu Kawasaki:** Conceptualization; data curation; investigation; writing – review and editing. **Yukihide Kanemitsu:** Conceptualization; data curation; investigation; writing – review and editing. **Yusuke Kinugasa:** Conceptualization; data curation; investigation; writing – review and editing. **Hideki Ueno:** Conceptualization; data curation; investigation; writing – review and editing. **Kotaro Maeda:** Conceptualization; data curation; investigation; writing – review and editing. **Takeshi Suto:** Conceptualization; data curation; investigation; writing – review and editing. **Michio Itabashi:** Conceptualization; data curation; investigation; writing – review and editing. **Kimihiko Funahashi:** Conceptualization; data curation; investigation; writing – review and editing. **Heita Ozawa:** Conceptualization; data curation; investigation; writing – review and editing. **Shingo Noura:** Conceptualization; data curation; investigation; writing – review and editing. **Hideyuki Ishida:** Conceptualization; data curation; investigation; writing – review and editing. **Masayuki Ohue:** Conceptualization; data curation; investigation; writing – review and editing. **Tomomichi Kiyomatsu:** Conceptualization; data curation; investigation; writing – review and editing. **Soichiro Ishihara:** Conceptualization; data curation; investigation; writing – review and editing. **Keiji Koda:** Conceptualization; data curation; investigation; writing – review and editing. **Hideo Baba:** Conceptualization; data curation; investigation; writing – review and editing. **Kenji Kawada:** Conceptualization; data curation; investigation; writing – review and editing. **Yojiro Hashiguchi:** Conceptualization; data curation; investigation; writing – review and editing. **Takanori Goi:** Conceptualization; data curation; investigation; writing – review and editing. **Yuji Toiyama:** Conceptualization; data curation; investigation; writing – review and editing. **Naohiro Tomita:** Conceptualization; data curation; investigation; writing – review and editing. **Eiji Sunami:** Conceptualization; data curation; investigation; writing – review and editing. **Fumihiko Fujita:** Conceptualization; data curation; investigation; writing – review and editing. **Jun Watanabe:** Conceptualization; data curation; investigation; writing – review and editing. **Kenichi Hakamada:** Conceptualization; data curation; investigation; writing – review and editing. **Goro Nakayama:** Conceptualization; data curation; investigation; writing – review and editing. **Kenichi Sugihara:** Conceptualization; data curation; funding acquisition; investigation; project administration; supervision; writing – review and editing. **Yoichi Ajioka:** Conceptualization; data curation; funding acquisition; investigation; project administration; supervision; writing – review and editing.

## CONFLICT OF INTEREST STATEMENT

Yusuke Kinugasa, Yuji Toiyama, Hideki Ueno, Takanori Goi, Jun Watanabe, and Kenichi Hakamada are editorial board members of Annals of Gastroenterological Surgery.

## ETHICS STATEMENT

Approval of research protocol: This study protocol was approved by the Japanese Society for Cancer of the Colon and Rectum and the ethical review boards of all 28 member hospitals who participated in this study.

Informed consent: Informed consent was obtained from all participants.

Registry and the registration No. of the study/trial: N/a.

Animal studies: N/a.
